# Gut microbiome–metabolome–ionome network spectrum mapping of colorectal cancer

**DOI:** 10.1016/j.gendis.2025.101566

**Published:** 2025-02-20

**Authors:** Xi Yang, Yin Jin, Yinhang Wu, Feng Zhou, Zhanbo Qu, Qing Zhou, Jiaying He, Ye Tao, Jing Zhuang, Shuwen Han

**Affiliations:** a Huzhou Central Hospital, Affiliated Central Hospital Huzhou University, Huzhou, Zhejiang 313000, China; b Huzhou Central Hospital, Fifth School of Clinical Medicine of Zhejiang Chinese Medical University, Huzhou, Zhejiang 313000, China; c Zhejiang-France United Laboratory of Integrated Traditional Chinese and Modern Medicine in Colorectal Cancer, Huzhou, Zhejiang 313000, China; d Hangzhou Black Box Biotechnology Co., Ltd., Hangzhou, Zhejiang 310020, China; e Shanghai Biozeron Biotechnology Co., Ltd, Shanghai 200000, China; f ASIR (Institute - Association of Intelligent Systems and Robotics), 14B rue Henri Sainte Claire Deville, Rueil-Malmaison 92500, France

Colorectal cancer (CRC) is one of the most common malignant tumors worldwide, and its occurrence and development are associated with a variety of factors,^1^ among which the roles of gut microbiota, metabolites, and ions are increasingly valued. However, the interaction relationship between intestine and intestinal microecology is complex, and it is difficult to explain the causal relationship between intestinal microbial interactions with current analytical methods. Structural equation modeling is a statistical modeling method for representing, estimating, and displaying a relationship network between variables.^2^^,^^3^ This method integrates factor analysis and path analysis to examine the complex relationships among multiple variables, thereby systematically elucidating the causal mechanisms underlying clinical phenomena. In this study, intestinal microbiome, metabolome, and ionome were detected in stool samples from healthy individuals and CRC patients, and multi-omics big data was used to map intestinal microecological composition. The structural equation model was used to construct the intestinal microbiome–metabolome–ionome network, and comprehensively illustrate the causal relationships among the complex and diverse influencing factors of CRC, providing a new perspective for revealing the pathogenesis of CRC.

The characteristics of the gut microbes, metal ions, and metabolites of 152 samples (83 healthy volunteers and 69 CRC patients) were analyzed (Table S1 and Fig. 1A, B). Detailed methods are provided in Supplementary File 1. Finally, 29 features, including 4 bacterial features (*CAG-180 sp000432435*, *E. coli_D, M. funiformis*, and *Prevotella* sp*900557255*), 6 bacterial functional enrichment features (biosynthesis of enediyne antibiotics, furfural degradation, isoflavonoid biosynthesis, nonribosomal peptide structures, photosynthesis-antenna proteins, and retrograde endocannabinoid signaling), 6 virus features (*Felixounavirus*, *Hpunavirus*, *Huchismacovirus*, *Peduovirus*, *Phikmvvirus*, and *Teseptimavirus*), 12 metabolome features (docosapentaenoic acid, hippuric acid, N4-acetylcytidine, glycocholic acid, thiamine, caffeine, trans-4-hydroxy-l-proline, yangonin, 2-phenylethylamine, methyldopa, fexofenadine, and adeninic acid), and 1 ionomic feature (titanium ion, Ti), were identified (Table S4).

**Figure 1:**
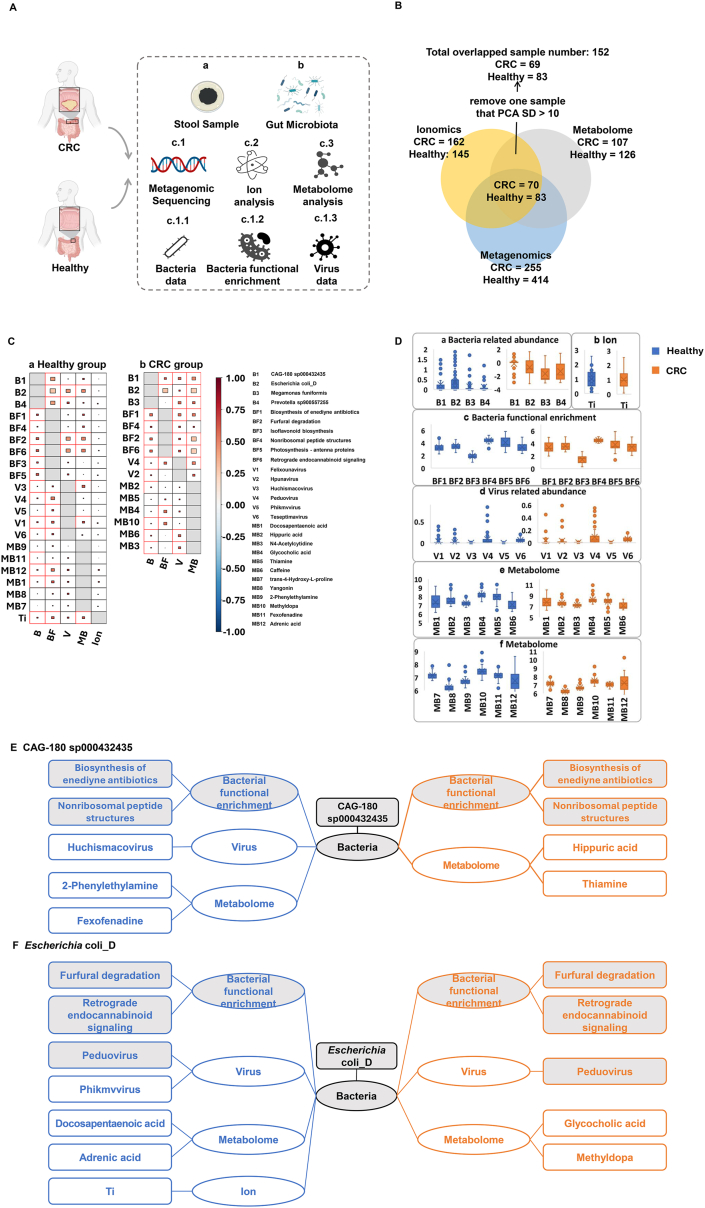
Outline description of the multiomics data and the similarity and difference in attribution analysis between the healthy and colorectal cancer (CRC) groups. **(A)** Flow diagram of sampling and analysis. **(B)** Samples tested in multi-omics. **(C)** Mental test between selected 29 features (21 in the normal group and 15 in the CRC group) with other omics data. The point in the red frame means *p* value < 0.05. **(D)** Boxplots of the selected 29 features in multi-omics. Boxplots of bacteria-related abundance data, economics data, bacterial functional enrichment data, virus data, and metabolome data after log10(x+1) changing. **(E, F)** Similarity and difference in attribution analysis between the normal and CRC groups. The characteristics in blue represent the features selected from the normal group, and those in orange represent the features selected from the CRC group. Characteristics with light gray backgrounds indicate the same features in the normal and CRC groups, and those with white backgrounds indicate different features in the normal and CRC groups.

Next, we made the mental test plot of the relationships among gut bacteria and related import features in viral, metabolomic, and ionomic data (Fig. 1C) as well as boxplots of the selected 29 features in multi-omics (Fig. 1D). Interestingly, each feature that was significantly correlated with the other omics data was positively correlated with both the normal (Table S5) and CRC (Table S6) groups. We found out that when CRC occurred, the relative abundance of *CAG-180 sp000432435* and the *E. coli_D* were reduced, though not significantly different from those in the healthy group (*p* > 0.05) (Fig. 1E, F).

Furthermore, we constructed a structural equation model^4^ to determine the contributions of the different features among multi-omics methods using the Python semopy package (https://pypi.org/project/semopy/) (Supplementary File 2), and combined the artificial intelligence algorithm and multi-omics to determine the causal relationships among gut microbes, metal ions, and metabolites. Finally, three bacteria (*CAG-180 sp000432435*, *Escherichia coli_D*, and *Prevotella* sp*900557255*) were selected each as the central feature and constructed structural equation models for the healthy group (Fig. S1 and Table S2). In the healthy group, *CAG-180 sp000432335* (Fig. S1A) had a large negative effect on both virus and metabolome and a positive effect on bacterial functional enrichment. Counterproductively, virus/metabolome and bacterial functional enrichment had medium and small negative effects on *CAG-180 sp000432335*, respectively. Virus and bacterial functional enrichment had negative effects on each other, and the same was true for metabolome and bacterial functional enrichment. Additionally, the virus had a positive effect on the metabolome, but the metabolome had a medium negative effect on the virus. *E. coli_D* (Fig. S1B) had small positive effects on the virus and bacterial functional enrichment, a medium positive effect on the virus and ionomics, and a small negative effect on the metabolome. Virus and metabolome had small negative and positive effects, respectively, on *E*. *coli_D*. The virus had negative and positive effects on metabolome and ionomics, respectively. Metabolome had the opposite effect on the virus. In addition, metabolome had a negative effect on bacterial functional enrichment. *P*. *sp900557255* (Fig. S1C) had medium negative effects on the virus and bacterial functional enrichment and a large positive effect on the metabolome. In turn, the virus, metabolome, and bacterial functional enrichment pathways had opposite effects. Virus, metabolome, and bacterial functional enrichment had opposite effects on each other. The effects of both virus and bacterial functional enrichment were negative.

In the CRC group, we selected three bacteria (*CAG-180 sp000432435*, *Escherichia coli_D*, and *Megamonas funiformis*) as the central feature and constructed structural equation models (Fig. S2 and Table S2). In the CRC group, *CAG-180 sp000432435* (Fig. S2A) had small and medium negative effects on the metabolome and bacterial functional enrichment, respectively, and the metabolome and bacterial functional enrichment had small negative and positive effects, respectively. The metabolome and bacterial functional enrichment had small negative effects on each other. *E. coli_D* (Fig. S2B) had a strong negative effect on the virus and small negative effects on both the metabolome and bacterial functional enrichment. Returning to *E. coli_D*, the virus and bacterial functional enrichment had negative effects but had large and medium effects, respectively, and the metabolome had a small positive effect. The bacterial functional enrichment had negative effects on the virus and metabolome, and *vice versa*. The virus had a positive effect on the metabolome, and the reverse had a negative effect. *M. uniforms* (Fig. S2C) had a small and medium negative effect on the metabolome and virus, respectively.

As we can see, *CAG-180 sp000432435* and *E. coli_D* have the same features. The bacterial functional enrichment features (biosynthesis of enediyne antibiotics) most strongly correlated with *CAG-180 sp000432435* were the same in the healthy and CRC groups (Fig. 1E). The top bacterial functional enrichment features (furfural degradation and retrograde endocannabinoid signaling) selected by *E. coli_D* were the same in the two groups, as was one virus feature, *Peduovirus* (Fig. 1F). However, some different features were selected for these two bacteria: for *CAG-180 sp000432435*, virus (*Huchismacovirus* in the healthy group) and metabolome (2-phenylethylamine and fexofenadine in the healthy group; hippuric acid and thiamine in the CRC group) were different; for *E. coli_D*, virus (*phikmvvirus* in the healthy group), metabolome (docosapentaenoic acid and adrenic acid in the healthy group; glycocholic acid and methyldopa in the CRC group), and ion (Ti in the healthy group) were different.

CRC alters the gut environment, which in turn affects the microbiome.^5^ The same factors interact differently in healthy individuals and those with CRC. For example, CAG-180 sp000432435 had positive effects on the biosynthesis of enediyne antibiotics and nonribosomal peptide structures in healthy individuals, but negative effects in those with CRC. *E. coli_D* facilitated furfural degradation in healthy individuals but exhibited an inhibitory effect on those with CRC. These phenomena may be attributed to changes in the gut microenvironment, leading to alterations in the ecological niches of different bacteria, and consequently, changes in various bacterial relationships.

The study has several limitations. A total of 152 subjects (83 volunteers and 69 CRC patients) were included, which may have resulted in an insufficient sample size. It is necessary to expand the sample size and conduct a multi-center study to further verify the reliability and universality of the findings. Moreover, the function of many bacteria can only be determined through statistical methods, and these functions need to be verified by subsequent cellular and animal experiments.

In short, we mapped the composition of gut microecology by multi-omics big data, and the interaction relationship network between microbes and related inorganic substances by structural equation model in CRC. In this way, comparing the relationship of *CAG-180 sp000432435* and *Escherichia coli_D* with viral and bacterial functions, ions, and metabolites between CRC patients and healthy individuals reveals that the causal relationships are not the same in CRC patients and healthy individuals.

## CRediT authorship contribution statement

**Xi Yang:** Conceptualization. **Yin Jin:** Data curation. **Yinhang Wu:** Writing – original draft. **Feng Zhou:** Data curation. **Zhanbo Qu:** Writing – original draft. **Qing Zhou:** Data curation. **Jiaying He:** Formal analysis. **Ye Tao:** Formal analysis. **Jing Zhuang:** Writing – original draft. **Shuwen Han:** Conceptualization.

## Ethics declaration

The patients' clinical protocols and informed consent were approved by the Ethics Committee of Huzhou Central Hospital (202202005-02) and the Chinese Clinical Trial Registry (http://www.chictr.org.cn, ChiCTR2100050167).

## Data availability

The datasets generated during the current study can be accessed from the China National GeneBank DataBase (CNGBdb), with the ID of CNP0004360.

## Funding

This work was supported by the Zhejiang Medical and Health Technology Project (China) (No. 2023KY1178, 2024KY408) and the Public Welfare Technology Application Research Program of Huzhou (Zhejiang Province, China) (No. 2024GY05).

## Conflict of interests

The authors declared no potential conflict of interests.
