# Supplementary material for: Gut microbiome–metabolome–ionome network spectrum mapping of colorectal cancer
Source: Genes Dis. 2025 Feb 20;13(1):101566. doi: 10.1016/j.gendis.2025.101566 (PMC12624594; doi:10.1016/j.gendis.2025.101566)
Supplement: Multimedia component 1 [file mmc1.doc]

**Materials and Methods**

**Subjects**

There were 669 samples (414 healthy volunteers and 255 CRC patients) for which the metagenomic data were sequenced, 307 samples (145 healthy volunteers and 162 CRC patients) that were tested using ionomics, and 233 samples (126 healthy volunteers and 107 CRC patients) that were tested using metabolomics. After removing one sample with a standard deviation (SD) > 10 in the bacterial principal component analysis (PCA), the number of overlapping samples that underwent all the abovementioned multiomics analyses was 152 (**Fig. 1A-B**; 83 healthy volunteers and 69 CRC patients). The 152 samples (**Table S1**) were subjected to downstream analysis. All the subjects provided signed informed consent under the guidance of the Ethics Committee of Huzhou Central Hospital. The patients' clinical protocols and informed consent were approved by the Ethics Committee of Huzhou Central Hospital (202202005-01) and the Chinese Clinical Trial Registry (http://www.chictr.org.cn, ChiCTR2100050167).

The inclusion criteria for patients were as follows: All CRC patients were confirmed by pathological diagnosis. The exclusion criteria for patients were as follows: had (1) other malignant tumors combined with (2) severe cardiopulmonary disease, (3) a history of antibiotic use within 3 months prior to admission, (4) a medical history of oral microbial agents within the last 2 months, or (5) other gut diseases, such as ulcerative colitis and Crohn's disease.

The specific clinical information of the volunteers involved in the data processing of this study came from the medical records management system of Huzhou Central Hospital.There were no significant differences in clinical factors, including sex, age, BMI, high blood pressure, hemameba, triglyceride, total cholesterol, glutamic-pyruvic transaminase, or glutamic oxalacetic transaminase (*P* > 0.05), between the normal and CRC groups.

Stool samples (approximately 5-10 grams) were collected without the use of laxatives or lubricants and stored in an ultralow temperature refrigerator within half an hour. The total storage time of the samples was less than 1 month.

**DNA Extraction and Sequencing**

High-quality DNA (OD260/280 = 1.8~2.2, OD260/230 ≥ 2.0) extracted from stool samples using the E.Z.N.A.® Viral DNA Kit (Omega Biotek, Norcross, GA, U.S.) was used to construct the sequencing library. The remaining supernatant was subjected to lysis, and viral DNA was extracted using the QIAamp Viral RNA Mini Kit without carrier RNA (Qiagen). Metagenomic shotgun sequencing libraries were constructed and sequenced at Shanghai Biozeron Biological Technology Co., Ltd. A Nextera XT DNA Library Preparation Kit from Illumina was used to construct sequencing libraries, and the concentrations of all the libraries were measured via a high-sensitivity double-stranded DNA kit on a Qubit fluorometer (Thermo Fisher Scientific). All the samples were sequenced on an Illumina NovaSeq instrument in paired-end (PE) 150 bp mode.

**Metagenomic read quality control and read-based phylogenetic annotation**

Adaptors and low-quality reads were trimmed from the raw data using Trimmomatic(http://www.usadellab.org/cms/uploads/supplementary/Trimmomatic)^[6]^. Clean reads were mapped against the human genome (NCBI) by the BWA mem algorithm (http://biobwa.sourceforge.net/bwa.shtml). The taxonomy of the clean reads for each sample was determined by the PathSeq pipeline distributed in GATK v4.1.3^[7]^ using the default database downloaded from the Broad Institute. PathSeq alignments were discarded if both read pairs did not match the same organism. All reads were classified into seven phylogenetic levels (domain, phylum, class, order, family, genus, species) or unclassified. The relationship between the host genome and phage was determined according to the annotations generated by PathSeq. The corresponding protein sequences were annotated via the KEGG website (https://www.kegg.jp/blastkoala/). The clean reads from each sample were aligned against the gene catalogs using BWA-MEM (v.0.7.17), and the abundance profiles of the genes were calculated as transcripts per million (TPM), with corrections for variations in gene length and mapped reads per sample. The relative abundance of the Kyoto Encyclopedia of Genes and Genomes (KEGG) pathway was calculated from the summation of the relative abundances of the genes included in the KEGG Orthologies (KOs).

**Metabolome** **detection and annotation**

Metabolomic data were obtained via the following steps. A 50 mg solid sample or 100 μL liquid sample was placed in a 1.5 mL centrifuge tube, and 400 μL of extraction solution (acetonitrile: methanol =1:1) was added. The solution was subjected to vortex mixing for 30 s and low-temperature ultrasonic extraction for 30 min (5℃, 40 KHz), placed at -20℃ for 30 min and then 4℃, and then centrifuged at 13000 g for 15 min. The supernatant was then removed, dried with nitrogen, and placed in 120 µL complex solution (acetonitrile: water = 1: 1). This solution was subject to redissolution, low-temperature ultrasonic extraction for 5 min (5℃, 40 KHZ), and centrifugation at 4℃ at 13000 g for 5 min, and then it was transferred into the injection vial with internal intubation for machine analysis. Metabolites from all the samples of equal volume were mixed to prepare quality control (QC) samples. In the process of instrumental analysis, a QC sample is inserted into every 10 samples to investigate the repeatability of the entire analysis process. The instrument platform for this LC‒MS analysis was AB SCIEX's ultrahigh-performance liquid chromatography tandem time-of-flight mass spectrometry (UPLC-TripleTOF) system.

After the computer was used, the raw LC‒MS data were imported into the metabolomics processing software Progenesis QI (Waters Corporation, Milford, USA) for baseline filtering, peak identification, integration, retention time correction, and peak alignment. Finally, a data matrix with retention time, mass-charge ratio and peak intensity was obtained. The data matrix uses the 80% rule to remove missing values; that is, variables with nonzero values above 80% are retained in at least one group of samples, after which the vacancy value is filled (the minimum value in the original matrix fills the vacancy value). To reduce the error caused by sample preparation and instrument instability, a data matrix was used to remove missing values. The response intensity of the sample essential spectrum peak is normalized by the sum normalization method, and the normalized data matrix is obtained. Moreover, the relative standard deviation (RSD) of the QC sample and gt was deleted; 30% of the variables were analyzed, and the log10 logization process was used to obtain the final data matrix for subsequent analysis. Moreover, the MS and MSMS mass spectrum information was matched with the public metabolic databases HMDB (http://www.hmdb.ca/) and Metlin (https://metlin.scripps.edu/) to obtain metabolite information.

The preprocessed data were uploaded to the Meggie Biocloud platform (https://cloud.majorbio.com) for data analysis. The R software package ropls (1.6.2) was used to perform PCA and orthogonal least partial squares discriminant analysis (OPLS-DA) and evaluate the stability of the model using 7 cycles of interactive validation. In addition, Student's t test and multiple difference analyses were performed. The selection of metabolites with significant differences was determined based on the variable weight value (VIP) obtained by the OPLS-DA model and the P value of Student's t test. A metabolite with a VIP > 1 and *P* < 0.05 was considered to be a significantly different metabolite. A total of 2,973 significantly differentially expressed metabolites were detected after screening, and with the help of the KEGG database (<https://www.kegg.jp/kegg/pathway.html>), the metabolic pathway annotation was completed. The Python software package scipy.stats was used for pathway enrichment analysis, and the biological pathways most relevant to the experimental treatment were identified by Fisher’s exact test.

**Ionomics detection**

We obtained the ionomic data by following the steps below. The samples were freeze-dried in a freeze-drying machine. The M (0.5 g, accurate to 0.001 g) sample was accurately weighed in a glass or Teflon digestion vessel. Then, the sample was placed an electric heating plate at a low temperature to remove ethanol or carbon dioxide, and 10 mL mixed solution of nitric acid (Sinopharm Group Chemical Reagent Co., LTD. Shanghai test 10014508) and perchloric acid (Tianjin Zhengcheng Chemical Products Co., LTD) (10:1) was placed on the electric heating plate to dissolve. After the digestion completed, the water was fixed to 50 mL. The instrument used was a Thermo Fisher iCAP 7200 HSDuo. The instrument test conditions were as follows: RF power, 1150 W; carrier gas flow rate, 0.7 L/min; auxiliary gas flow rate, 1.0 L/min; cooling gas flow rate, 12.0 L/min; and detection mode: axial. A calibration curve was drawn by mixing standard materials (32 kinds of mixed metal labels; tan Ink quality inspection number: BWT30121-100-100; batch number: B22120033) and assigning 5 mass concentration points: 0 mg/L, 2 mg/L, 5 mg/L, 10 mg/L, and 20 mg/L. The calculation is as follows:


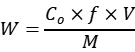


*W* is the final test result of the measured element and is calculated by the above formula, in mg/kg;
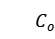
 is the test solution element concentration, and the data obtained by the instrument test are in mg/L. *f* indicates the dilution ratio. *M* represents the mass of the sample taken, in g; *V* represents the volume of constant volume after sample digestion in mL.

**Structural equation models**

We used the Python scikit-learn package^[8]^ to select bacteria that exhibited the most variation within the normal or CRC group. After bacterial selection, we used the Python script package^[9^**]** to analyze the Pearson correlation between the bacteria and other omics features and obtained the topmost correlated features in the normal and CRC groups. With the R package vegan^[10]^, we analyzed the relationships between these selected features and different omics data based on the Mantel test in the normal and CRC groups. To normalize the dimensions, we convert all the data with a 0-1 transformation using the following formula:


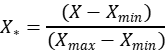


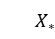
 is the normalized feature value ∈ (0, 1), *X* is the raw feature value,
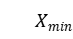
 is the sample minimum value of this feature, and
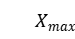
 is the sample maximum value of this feature. We use the 0–1-transformed values for the following analysis. Moreover, we constructed structural equation models ^[11]^ to determine the contributions of the different features among multiomics methods using the Python semopy package (**Suppement File 1**) ^[5]^. *P* value of chi-square test (chi2 p-value), comparative fit index (CFI), goodness of fit (GFI), normed fit index (NFI) and root mean square error of approximation (RMSEA) were used to evaluate the effectiveness of the model. The higher the chi2 p-value (more little difference between the expected and observed covariance matrices), CFI, GFI, and NFI, and the lower the RMSEA, the better model fit ^[12]^.

**References**

[6]Bolger, A. M., M. Lohse, et al. (2014). "Trimmomatic: a flexible trimmer for Illumina sequence data." Bioinformatics 30(15): 2114-2120.

[7]Kostic AD, Ojesina AI, Pedamallu CS, Jung J, Verhaak RG, Getz G, Meyerson M. (2011) PathSeq: software to identify or discover microbes by deep sequencing of human tissue. Nat Biotechnol. May;29(5):393-6. doi: 10.1038/nbt.1868. PMID: 21552235; PMCID: PMC3523678.

[8]https://scikit-learn.org/stable/modules/feature_selection.html.

[9]https://docs.scipy.org/doc/scipy/reference/stats.html.

[10]https://www.rdocumentation.org/packages/vegan/versions/2.6-4.

[11]Maruyama, G. M. (1998). Basics of structural equation modeling. SAGE Publications, Inc., <https://doi.org/10.4135/9781483345109>.

[12]Suhr D. The basics of structural equation modeling[J]. Presented: Irvine, CA, SAS User Group of the Western Region of the United States (WUSS), 2006.
