# Supplementary material for: Gut microbiome–metabolome–ionome network spectrum mapping of colorectal cancer
Source: Genes Dis. 2025 Feb 20;13(1):101566. doi: 10.1016/j.gendis.2025.101566 (PMC12624594; doi:10.1016/j.gendis.2025.101566)
Supplement: Multimedia component 3 [file mmc3.doc]

**Table S1 Clinical information of the patients included in this study.**

| Clinical factors | Normal group  (N = 83) | CRC group  (N = 69) | *P* value |
| --- | --- | --- | --- |
| Gender |  |  | 0.85 |
| Female | 47 (56.6%) | 38 (55.1%) |  |
| Male | 36 (43.4%) | 31 (44.9%) |  |
| Age (years) | 61.94±10.19 | 64.35±9.52 | 0.14 |
| BMI | 15.10±3.71 | 27.07±9.38 | 0.11 |
| High blood pressure | 19 (22.9%) | 22 (31.9%) | 0.21 |
| Hemameba (×10^9^/L) | 5.89±1.31 | 6.07±2.30 | 0.07 |
| Albumin (g/L) | 38.49±4.27 | 37.71±4.02 | 0.25 |
| Triglyceride (mmol/L) | 1.52±0.71 | 1.28±0.86 | 0.07 |
| Total cholesterol (mmol/L) | 4.74±1.29 | 4.46±1.09 | 0.15 |
| Glutamic-pyruvic transaminase (U/L) | 21.18±9.70 | 20.98±13.02 | 0.91 |
| Glutamic oxalacetic transaminase (U/L) | 23.65±6.87 | 24.01±10.43 | 0.80 |
