# Supplementary material for: Gut microbiome–metabolome–ionome network spectrum mapping of colorectal cancer
Source: Genes Dis. 2025 Feb 20;13(1):101566. doi: 10.1016/j.gendis.2025.101566 (PMC12624594; doi:10.1016/j.gendis.2025.101566)
Supplement: Multimedia component 4 [file mmc4.doc]

**Table S2 Pearson correlation (*P* < 0.001) of the top variant bacteria in the normal group with multiomics data**

| Bacteria | Features in other omics | r | *P* value |
| --- | --- | --- | --- |
| CAG-180 sp000432435 | Biosynthesis of enediyne antibiotics | 0.978 | **< 0.001** |
|  | Nonribosomal peptide structures | 0.684 | **< 0.001** |
|  | 2-Phenylethylamine | 0.507 | **< 0.001** |
|  | Fexofenadine | 0.395 | **< 0.001** |
|  | *Huchismacovirus* | 0.470 | **< 0.001** |
| *Escherichia* coli_D | Furfural degradation | 0.992 | **< 0.001** |
|  | Retrograde endocannabinoid signaling | 0.982 | **< 0.001** |
|  | Ti | 0.462 | **< 0.001** |
|  | Adrenic acid | 0.713 | **< 0.001** |
|  | Docosapentaenoic acid | 0.584 | **< 0.001** |
|  | *Peduovirus* | 0.642 | **< 0.001** |
|  | *Phikmvvirus* | 0.424 | **< 0.001** |
| *Prevotella* sp900557255 | Isoflavonoid biosynthesis | 0.680 | **< 0.001** |
|  | Photosynthesis–antenna proteins | 0.843 | **< 0.001** |
|  | Yangonin | 0.495 | **< 0.001** |
|  | trans-4-Hydroxy-L-proline | 0.420 | **< 0.001** |
|  | *Felixounavirus* | 0.649 | **< 0.001** |
|  | *Teseptimavirus* | 0.512 | **< 0.001** |

Note that the characters in bold indicate significant differences.
