# Supplementary material for: Gut microbiome–metabolome–ionome network spectrum mapping of colorectal cancer
Source: Genes Dis. 2025 Feb 20;13(1):101566. doi: 10.1016/j.gendis.2025.101566 (PMC12624594; doi:10.1016/j.gendis.2025.101566)
Supplement: Multimedia component 5 [file mmc5.doc]

**Table S3 Pearson correlation (*P* < 0.001) of the top variant bacteria in the CRC group with multiomics data**

| Bacteria | Features in other omics | r | *P* value |
| --- | --- | --- | --- |
| CAG-180 sp000432435 | Biosynthesis of enediyne antibiotics | 0.937 | **< 0.001** |
|  | Nonribosomal peptide structures | 0.573 | **< 0.001** |
|  | Hippuric acid | 0.608 | **< 0.001** |
|  | Thiamine | 0.552 | **< 0.001** |
| *Escherichia* coli_D | Furfural degradation | 0.930 | **< 0.001** |
|  | Retrograde endocannabinoid signaling | 0.949 | **< 0.001** |
|  | Glycocholic acid | 0.789 | **< 0.001** |
|  | Methyldopa | 0.809 | **< 0.001** |
|  | *Peduovirus* | 0.657 | **< 0.001** |
| *Megamonas* funiformis | Caffeine | 0.917 | **< 0.001** |
|  | N4-Acetylcytidine | 0.765 | **< 0.001** |
|  | *Hpunavirus* | 0.905 | **< 0.001** |

Note that the characters in bold indicate significant differences.
