# Supplementary material for: Gut microbiome–metabolome–ionome network spectrum mapping of colorectal cancer
Source: Genes Dis. 2025 Feb 20;13(1):101566. doi: 10.1016/j.gendis.2025.101566 (PMC12624594; doi:10.1016/j.gendis.2025.101566)
Supplement: Multimedia component 6 [file mmc6.doc]

**Table S4 One-way ANOVA of the 29 selected features among the normal and CRC groups**

| Omics | Feature | F value | *P* value |
| --- | --- | --- | --- |
| Bacteria | CAG-180 sp000432435 | 1.111 | 0.293 |
|  | *Escherichia* coli_D | 1.536 | 0.217 |
|  | *Megamonas* funiformis | 0.921 | 0.338 |
|  | *Prevotella* sp900557255 | 1.925 | 0.167 |
| Bacterial functional enrichment | Biosynthesis of enediyne antibiotics | 1.228 | 0.270 |
|  | Furfural degradation | 1.993 | 0.1601 |
|  | Isoflavonoid biosynthesis | 12.195 | **< 0.001** |
|  | Nonribosomal peptide structures | 1.377 | 0.242 |
|  | Photosynthesis–antenna proteins | 1.849 | 0.176 |
|  | Retrograde endocannabinoid signaling | 1.302 | 0.256 |
| Virus | *Felixounavirus* | 1.03E-05 | 0.997 |
|  | *Hpunavirus* | 1.201 | 0.275 |
|  | *Huchismacovirus* | 0.369 | 0.545 |
|  | *Peduovirus* | 0.090 | 0.764 |
|  | *Phikmvvirus* | 3.624 | 0.059 |
|  | *Teseptimavirus* | 1.250 | 0.265 |
| Metabolome | Docosapentaenoic acid | 4.496 | **0.036** |
|  | Hippuric acid | 0.631 | 0.428 |
|  | N4-Acetylcytidine | 0.024 | 0.878 |
|  | Glycocholic acid | 2.526 | 0.114 |
|  | Thiamine | 0.014 | 0.907 |
|  | Caffeine | 1.150 | 0.285 |
|  | trans-4-Hydroxy-L-proline | 0.531 | 0.467 |
|  | Yangonin | 0.672 | 0.414 |
|  | 2-Phenylethylamine | 0.959 | 0.329 |
|  | Methyldopa | 1.529 | 0.218 |
|  | Fexofenadine | 5.818 | **0.017** |
|  | Adrenic acid | 1.716 | 0.192 |
| Ionomics | Ti | 0.117 | 0.732 |

Note that the characters in bold indicate significant differences.
