# Supplementary material for: Gut microbiome–metabolome–ionome network spectrum mapping of colorectal cancer
Source: Genes Dis. 2025 Feb 20;13(1):101566. doi: 10.1016/j.gendis.2025.101566 (PMC12624594; doi:10.1016/j.gendis.2025.101566)
Supplement: Multimedia component 10 [file mmc10.pdf]

Fig. S2. Structural equation models (SEMs) of the 3 top variant bacteria in the CRC samples.

The data were normalized on a 0-1 scale to unify dimensions among different omics levels. A-C. *CAG-180 sp000432435, Escherichia coli_D*, and *Megamonas funiformis* attribution analysis. a. SEM. If the path is bidirectional, then the colorful arrow is used; otherwise, the single arrow is used. The values on the arrows indicate the capacity of the path, path coefficients with absolute values < 0.1 indicate a "small" effect, values approximately 0.3 indicate a "medium" effect, and values > 0.5 indicate a "large" effect. ns: *P* value > 0.05, *: *P* value < 0.05; **: *P* value < 0.01, ***: *P* value < 0.001; the significant paths are not shown in the figures. The arrow points from one feature and goes back to itself, indicating the variance of that feature. b. The correlation curve between the bacteria and other omics data was strongly correlated with the bacteria. c. Fitting of the related SEMs. chi2: chi-square value, chi2 p value: p value of chi-square test, CFI: comparative fit index, GFI: goodness of fit, NFI: normed fit index, RMSEA: root mean square error of approximation.

A CAG-180 sp000432435

a

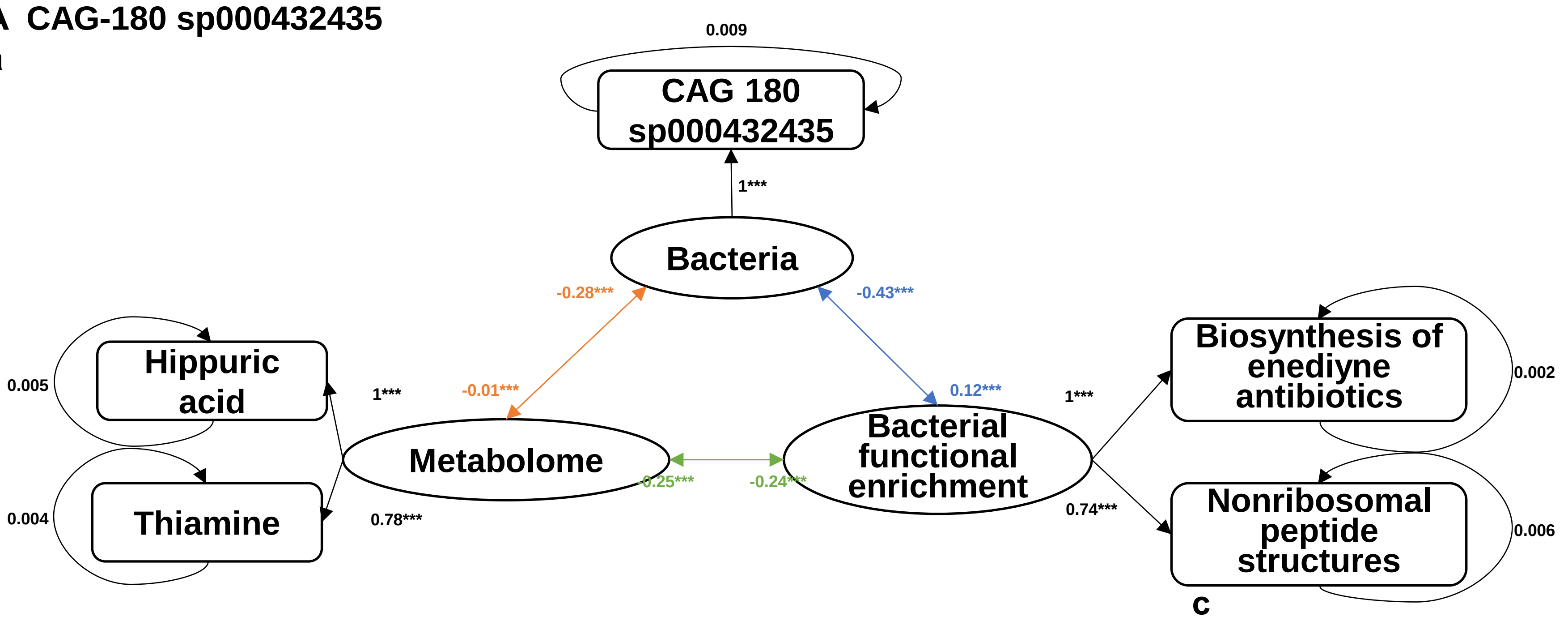

b

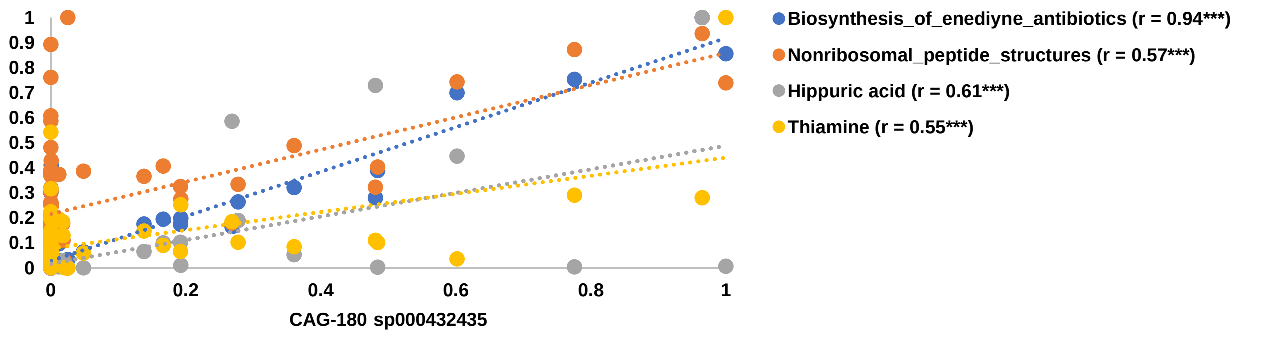

c

Index	Value
chi2	3.58
chi2 p-value	>0.05
CFI	0.97
GFI	0.99
NFI	0.99
RMSEA	0

B *Escherichia* coli_D

a

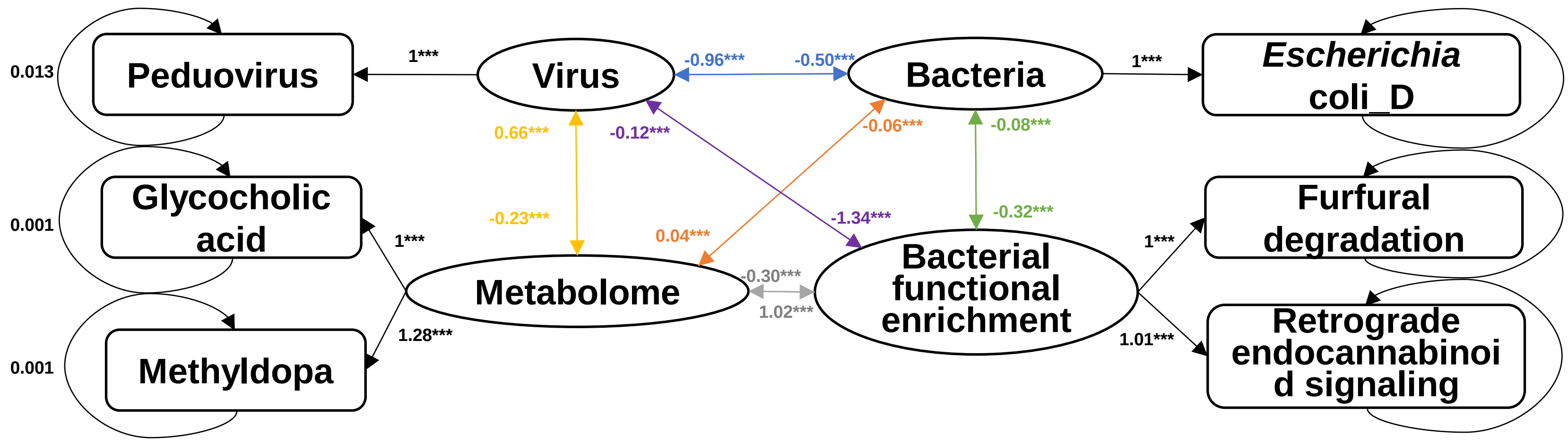

b

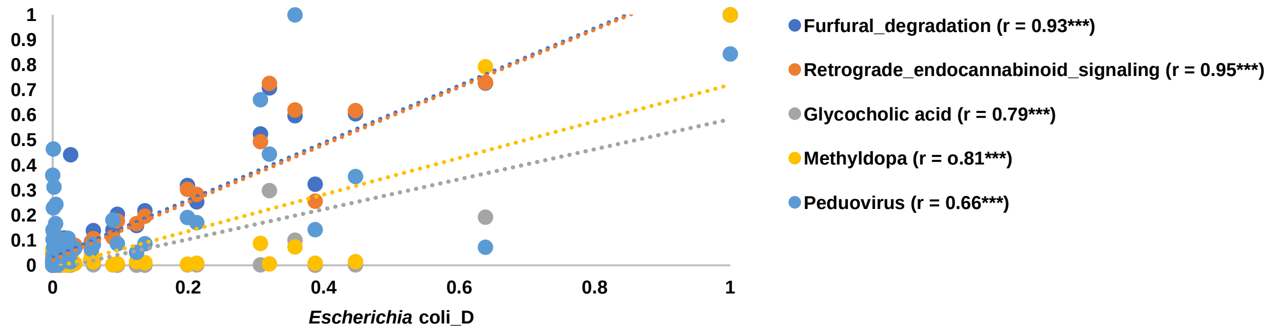

c

Index	Value
chi2	11.77
chi2 p-value	>0.05
CFI	0.96
GFI	0.98
NFI	0.98
RMSEA	0

C *Megamonas funiformis*

a

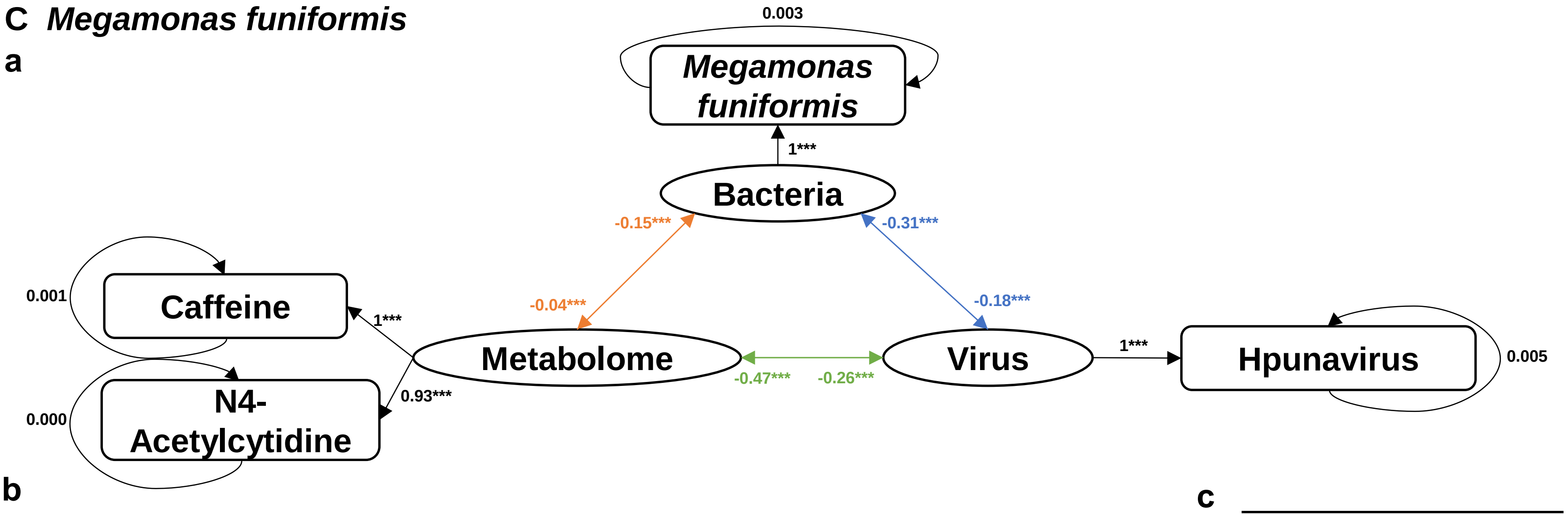

b

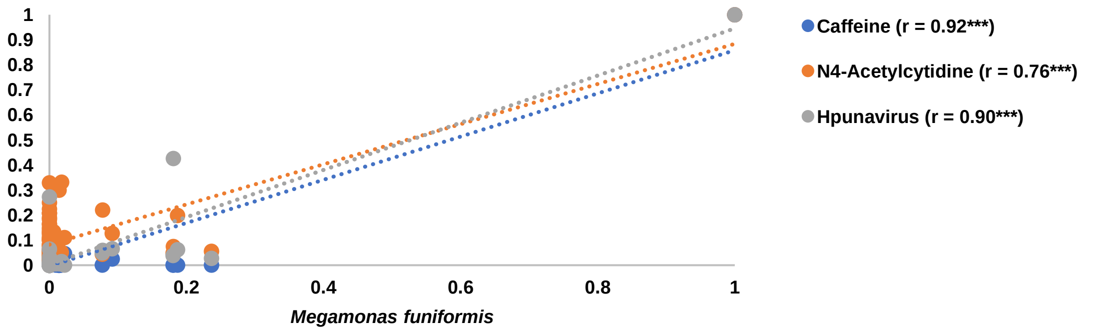

c

Index	Value
chi2	0.01
chi2 p-value	>0.05
CFI	0.98
GFI	1.00
NFI	1.00
RMSEA	0
